# Weekly cisplatin and daily oral etoposide is highly effective in platinum pretreated ovarian cancer

**DOI:** 10.1038/sj.bjc.6600002

**Published:** 2002-01-07

**Authors:** M E L van der Burg, R de Wit, W L J van Putten, A Logmans, W H J Kruit, G Stoter, J Verweij

**Affiliations:** Department of Medical Oncology, Rotterdam Cancer Institute and University Hospital, Rotterdam, P.O. Box 5201, 3008 AE Rotterdam, The Netherlands; Department of Gynecology, Rotterdam Cancer Institute and University Hospital, 3008 AE Rotterdam, The Netherlands; Biostatistics, Rotterdam Cancer Institute and University Hospital, 3008 AE Rotterdam, The Netherlands

**Keywords:** ovarian cancer, chemotherapy, cisplatin, etoposide

## Abstract

We investigated the potential of weekly cisplatin and daily oral etoposide followed by oral etoposide maintenance therapy in patients with platinum-refractory ovarium cancer. One hundred and seven patients were entered on the study, 98 patients completed the induction therapy consisting of cisplatin at either 50 or 70 mg m^−2^ weekly for six administrations plus oral etoposide at a dose of 50 mg daily. Of these 98 patients, 38 had a platinum treatment-free interval of more than 12 months, 32 had an interval between 4 and 12 months, and 28 had progressed during or within 4 months after last platinum therapy. We assessed response rates and time to progression, and also response duration and survival. Analyses were done on the 98 evaluable patients. All 107 patients were considered evaluable for toxicity. Of the 38 patients with a treatment-free interval of more than 12 months, 92% responded, with 63% complete responses. The median progression-free survival in these patients was 14 months, and the median survival was 26 months. Of the 32 patients with an interval of 4–12 months, 91% responded, with 31% complete responses, a median progression-free interval of 8 and a median overall survival of 16 months. Of the 28 patients with platinum-refractory disease, 46% as yet responded, with 29% complete responses, median progression-free interval of 5 and an overall survival of 13 months. Haematologic and non-haematologic, particularly renal toxicity and neurotoxicity, were notably mild. We conclude that this intensive regimen of weekly cisplatin plus daily etoposide is highly effective and well tolerated in patients with ovarian cancer relapsing after conventional platinum-based combination chemotherapy, including patients who have progressed during or within 4 months after platinum treatment.

*British Journal of Cancer* (2002) **86**, 19–25. DOI: 10.1038/sj/bjc/6600002 www.bjcancer.com

© 2002 The Cancer Research Campaign

## 

Despite the established role of platinum-based combination chemotherapy in the treatment of ovarian cancer, resulting in prolonged disease-free survival even in patients with overt metastatic disease, unfortunately the majority of patients eventually will relapse (Neijt *et al*, 1991).

Although reinduction therapy with platinum compounds may again produce responses, durable survival is rare. Multiple studies have shown that the time interval between the completion of first-line chemotherapy and the relapse is the most important prognostic factor in the likelihood of responding to second-line therapy (Seltzer *et al*, 1985; Gershenson *et al*, 1989; Kavanagh and Nicaise, 1989; Markman *et al*, 1991; van der Burg *et al*, 1991; Kjorstad *et al*, 1992; Berek *et al*, 1998). This impact accounts not only for the successful reinduction rate to platinum but also for the response rate to other active agents, including the taxanes, topotecan, gemcitabine and anthracyclins (de Wit *et al*, 1994; Eisenhauer *et al*, 1994; Lund *et al*, 1994; Kavanagh *et al*, 1996; Muggia *et al*, 1997; ten Bokkel Huinink *et al*, 1997; Rose *et al*, 1998). In patients who relapse within 4 months following first-line therapy, usually defined as platinum-refractory disease, the response rate to any conventional platinum regimen as well as to other compounds is less than 15%. In patients who relapse between 4 and 12 months following therapy, defined as intermediate platinum-sensitive disease, responses are obtained in 20–30% of patients, and are usually of brief duration. Only if relapses occur more than 12 months after first line treatment, 50% of the patients will again benefit from platinum-containing reinduction chemotherapy, which can be considered standard of care.

In view of the observed dose–response relationship for platinum compounds in *in-vitro* ovarian cancer models, dose-intensive therapy has been investigated in phase II studies (Ozols *et al*, 1987). An alternative strategy to increase the dose of cisplatin administered over a given period of time, is to reduce the interval between cycles. We have previously shown that by inducing a chloro-uresis through dissolving cisplatin in 3% hypertonic saline, cisplatin at a dose of 70 mg m^−2^ weekly can be safely administered in platinum-naive patients with solid tumours (Planting *et al*, 1993). In view of the observed synergy between cisplatin and etoposide (Mabel, 1979; Chambers *et al*, 1987) and the activity of prolonged oral administration of etoposide, (de Wit *et al*, 1994; Rose *et al*, 1998) we subsequently investigated combined weekly cisplatin with daily etoposide (Planting *et al*, 1995). We presently report on a phase II study with this new modality of weekly cisplatin and daily oral etoposide in patients failing conventional platinum-based combination chemotherapy.

## PATIENTS AND METHODS

### Patients

Eligibility required histologically confirmed epithelial ovarian cancer (Brenner tumours and borderline tumours were excluded), progressive disease or relapse after at least one platinum combination regimen, WHO performance status 0–2, bidimensional target lesion measurable disease by radiographic, gynaecological or physical examination. Pretreatment laboratory requirements included WBC ⩾3.0×10^9^ l^−1^, platelets ⩾100×10^9^ l^−1^, serum bilirubin ⩽25 μmol l^−1^, serum creatinine ⩽120 μmol l^−1^ and/or a creatinine clearance ⩾60 ml min^−1^. Exclusion criteria were peripheral neuropathy from previous therapy Common Toxicity Criteria (CTC) ⩾grade 2, or bowel obstruction. At the initiation of the study only the platinum-sensitive patients (recurrence >12 months after last platinum chemotherapy) were eligible. When this regimen of weekly cisplatin and daily etoposide showed to be both effective and feasible, the study protocol was amended to include all patients with progression during or within 12 months after platinum chemotherapy. The protocol was approved by the Institutional Ethics Board. All patients gave written informed consent.

### Treatment

The treatment regimen consisted of 6 weekly i.v. cisplatin infusions on day 1, 8, 15 and day 29, 36, 43, combined with daily oral etoposide 50 mg on days 1–15 and days 29–43. The cisplatin dose was 50 mg m^−2^ in the platinum-sensitive patients, and later during the conduct of the study 70 mg m^−2^ in the intermediate-sensitive and -refractory patients. Patients with a response or stable disease after the sixth cisplatin administration continued treatment with oral etoposide 50 mg m^−2^ per day for 21 days, every 4 weeks, for 6–9 cycles.

Cisplatin was dissolved in 250 ml NaCl 3% and administered over 3 h. The cisplatin infusion started after prehydration with 1000 ml dextrose-saline with 20 mmol KCl and 2 g MgSO_4_. After the cisplatin infusion the patients received posthydration consisting of 2 liters dextrose-saline with 40 mmol KCl and 4 g MgSO_4_ given over 8 h. All patients received ondansetron 8 mg and dexamethasone 10 mg i.v. 30 min before the start of cisplatin.

### Dose reductions

#### Weekly cisplatin and daily etoposide

If on day 8 or day 36 WBC was <2.5×10^9^  l^−1^ and/or platelets <75×10^9^  l^−1^, treatment was postponed 1 week until recovery. If on day 15 or 43 WBC was <1.5×10^9^ l^−1^ and/or platelets <50×10^9^ l^−1^ the cisplatin infusion was omitted. If on day 29 WBC was <3.0×10^9^ l^−1^ and/or platelets <100×10^9^ l^−1^, the treatment was postponed 1 week until recovery. Cisplatin administration was ceased in case the creatinine clearance fell below 45 ml min^−1^ or in case of neurotoxicity grade ⩾3.

### Etoposide monotherapy

If WBC was <3.0×10^9^ l^−1^ and/or platelets <100×10^9^ l^−1^ on day 1, the treatment was postponed 1 week until recovery. If on day 8 or day 15 WBC was <2.0×10^9^ l^−1^ and/or platelets <50×10^9^ l^−1^, the cycle was discontinued.

### Treatment monitoring

During treatment, weekly blood counts, serum electrolytes, renal and liver function tests were performed. Response to treatment was assessed by physical and gynaecological examination, vaginal ultrasound and CT scan on day 56 and every 3 months thereafter. In case of discrepancy in response assessment, response was determined by the study team. The worst response was then taken into account for the analysis. A senior staff member of the Institute who was not involved in the conduct of the study reviewed all responses. Responses were determined according to World Health Organization (WHO) criteria (WHO, 1979).

### Safety parameters

Quantitative haematologic and non-haematologic toxicities were assessed according to the National Cancer Institute (NCI) common toxicity criteria (CTC). For the assessment of toxicity, the patients were grouped according to the cisplatin dose of 50 and 70 mg m^−2^ weekly, respectively.

### Statistical methods

Main endpoints for the analysis were response, response duration, progression-free survival (PFS), overall survival (OS) and toxicity. Response duration, PFS and OS were calculated by actuarial methods. The response duration and PFS was defined as the interval between the start of treatment and progression. The OS was defined as the interval between the start of therapy and death or last follow-up of all evaluable patients, with censoring of the patients alive at last follow-up. The method of Kaplan and Meier was used to calculate median survival times and probabilities at 1 and 2 years after start of treatment. The Cox regression model was used to test for differences in PFS and OS between subgroups defined by platinum sensitivity, performance status, histology, tumour size, the presence of ascites, and the number of previous chemotherapy regimens.

For the response and survival evaluations the patients were grouped according to the definition of the recent consensus classification (Berek *et al*, 1998); patients with an interval less than 4 months between progression and the last platinum-based chemotherapy were defined platinum-refractory, an interval between 4–12 months was defined intermediate-sensitive, and an interval of more than 12 months was defined platinum-sensitive.

Patients were considered evaluable for response if they had completed the induction therapy of the weekly administrations of cisplatin plus daily etoposide. Patients were evaluable for toxicity if they had received at least one administration of cisplatin.

## RESULTS

### Patient characteristics

One hundred and seven patients were entered onto the study. One patient was ineligible since she has no measurable or evaluable disease. Eight patients were not evaluable for response since they did not complete the induction therapy. Reasons were patient refusal after 1–3 cycles (5) abdominal aneurysm (1) and myocardial infarction (1), ileus after one cycle (1). The patient characteristics of the 98 patients who were eligible and evaluable for response are presented in Table 1

**Table 1:**
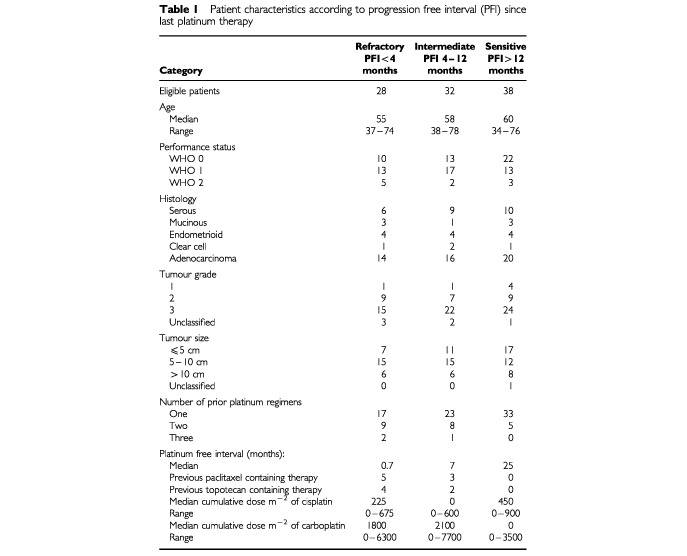
Patient characteristics according to progression free interval (PFI) since last platinum therapy

.

The greater majority of patients had been previously treated with one or two regimens of cisplatin plus cyclophosphamide or carboplatin plus cyclophosphamide between 1994 and 1997 when platinum-paclitaxel combination chemotherapy had not yet become standard induction therapy. The mean cumulative cisplatin dose per m^2^ at enrollment of the study was 306 mg (s.d. 264). The mean cumulative carboplatin dose per m^2^ at enrollment was 1328 mg (s.d. 1527). A total of eight patients had previously been treated with paclitaxel containing chemotherapy, and six patients had been treated with topotecan.

The median duration of follow-up was 13 months for platinum-refractory patients (= median survival), 16 months for intermediate-sensitive patients (= median survival), and 26 months for platinum-sensitive patients.

### Toxicity of weekly cisplatin and daily etoposide

All 107 patients were considered evaluable for toxicity. The haematologic and non-haematologic toxicity by dose of cisplatin is listed in Table 2

**Table 2:**
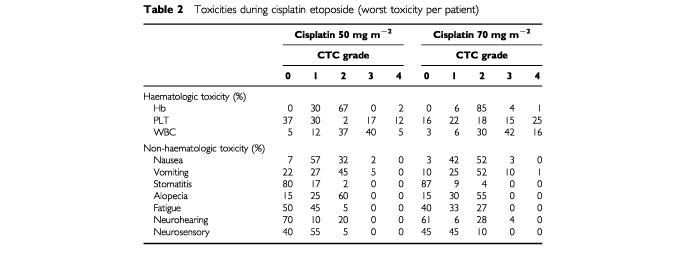
Toxicities during cisplatin etoposide (worst toxicity per patient)

. Forty patients received a total of 263 administrations at a cisplatin dose of 50 mg m^−2^, and 67 patients received 358 administrations at a dose of 70 mg m^−2^. Myelotoxicity was acceptable with no more than one case of neutropenic fever. A number of patients, five on 50 mg m^−2^, and 17 on 70 mg m^−2^, had grade 4 trombocytopenia, but there were no haemorrhages. As a result of the blood cell counts on days 29, and nadir counts on days 36 and 43, in 40% of the patients treatment was postponed once by 1 week, and in 10% twice.

Non-haematologic toxicity was notably mild. At the start of study treatment, 31 patients had grade 1 and four patients had grade 2 neurotoxicity resulting from the prior platinum therapy.

After completion of the weekly induction therapy an additional 21 patients had acute grade 1 neurotoxicity and one patient had grade 2 neurotoxicity, whereas neurotoxicity had not clinically significantly worsened in the patients with preexisting neuropathy. No neuromotor toxicity was observed.

Late neurotoxicity, evaluated 6 months after the completion of the weekly cisplatin cycles accounted for a total of 22 patients with grade 1 symptoms and six patients with grade 2 symptoms. Renal toxicity was mild. Renal toxicity grade 1 and 2 was observed in 4% of the patients receiving cisplatin at the dose of 70%. The highest measured creatinine value was 165 μmol. After 6 months follow-up one patient still had grade 2 nephrotoxicity.

### Toxicity of etoposide monotherapy

Of the 98 patients who completed the weekly cisplatin and oral etoposide treatment, 90 patients continued with oral etoposide; 20 patients in the platinum-refractory group continued for a median of three cycles (range, 1–9 cycles), 32 patients in the intermediate-sensitive group for a median of three cycles (range, 1–9 cycles), and 38 platinum-sensitive patients for a median of six cycles (range, 1–9 cycles).

Haematologic toxicity during the maintenance therapy with daily oral etoposide rarely resulted in grade 4 toxicity (2%). There was one fatal case of neutropenic sepsis. Non-haematologic toxicity of etoposide was generally mild, with fatigue, mostly related to treatment related anaemia and grade 1 or 2 stomatitis in 4%.

### Dose intensity achieved

The planned dose intensity of six administrations of cisplatin, calculated over a treatment period of 8 weeks, was 37.5 mg m^−2^ week^−1^ for the 50 mg m^−2^ patients and 52.5 mg m^−2^ week^−1^ for the 70 mg m^−2^ patients. The achieved median dose intensity was 32 mg m^−2^ week^−1^ (range 21–38 mg m^−2^ week^−1^) and 45 mg m^−2^ week^−1^ (range 21–53 mg m^−2^ week^−1^), respectively. The median dose-intensity achieved with oral etoposide maintenance therapy was 41 mg m^−2^ week^−1^ (range 21–51 mg m^−2^ week^−1^).

### Response and survival

At the time of completion of the weekly cisplatin and daily etoposide there were four complete responses (CR) and seven partial responses (PR) in the 28 platinum-refractory patients (39%), three CR and 26 PR in the 32 intermediate-sensitive patients (91%), and five CR and 24 PR in the 38 platinum-sensitive patients (76%). Of the 38 platinum-sensitive patients, 14 underwent interval surgery, which confirmed four CR, one clinical CR patient had minimal residual disease, and in four patients with a PR residual tumour was debulked to less than 1 cm.

During and at the time of completion of daily etoposide maintenance therapy in responding and stable disease (SD) patients, several PR's converted into CR and SD into PR. The overall response to the protocol treatment is shown in Table 3

**Table 3:**
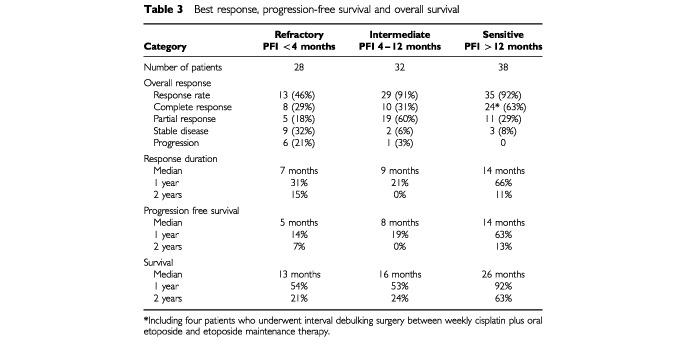
Best response, progression-free survival and overall survival

. The overall response rate to weekly cisplatin plus daily etoposide and continued treatment with daily oral etoposide was as high as 92% in platinum-sensitive patients, 91% in intermediate-sensitive patients, and 46% in refractory patients.

**Figure 1:**
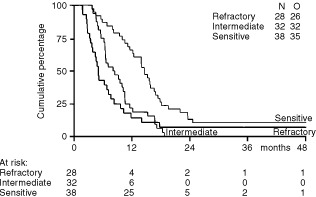
PFS according to platinum sensitivity.

Within the group of patients with platinum-sensitive tumours who had a 92% overall response rate, 24 of 38 (63%) of patients obtained a CR. The median response duration in this group was 14 months, the median PFS was 14 months and the median OS was 26 months. Although the 32 patients in the intermediate group also had a high overall response rate of 91%, the CR rate was lower, 10 of 32 (31%), compared with that observed in the sensitive group. Also, the median response duration of 9 months, the median PFS of 8 months and the median OS of 16 months were less than were observed in the sensitive group. Of the 28 patients who were considered cisplatin-refractory, 13 (46%) still responsed to weekly cisplatin plus daily etoposide reinduction therapy and daily oral etoposide maintenance therapy, eight of whom (29%) obtained a CR. The median response duration in the refractory patient group was 7 months, the median PFS was 5 months, and the median OS was 13 months. Response durations, PFS and OS in the three groups are shown in Table 3. Figures 1

and 2

**Figure 2:**
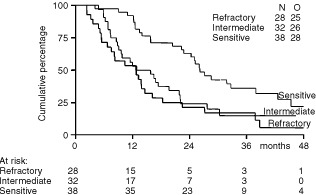
Survival according to platinum sensitivity.

show the curves for PFS and OS in the three groups.

### Prognostic factors

The median PFS and OS in patients with a WHO performance of zero was 12 and 23 months, respectively, *vs* 7 and 13 months, respectively, in patients with WHO performance status 1–2. In patients with tumour lesions less than 5 cm the median PFS and OS was 14 and 25 months, respectively, *vs* 6 and 13 months, respectively, in patients with larger lesions.

In the univariate analysis, progression-free interval (PFI) since the last platinum-based chemotherapy, performance status and tumour size were significant prognostic factors both for PFS and OS (Table 4

**Table 4:**
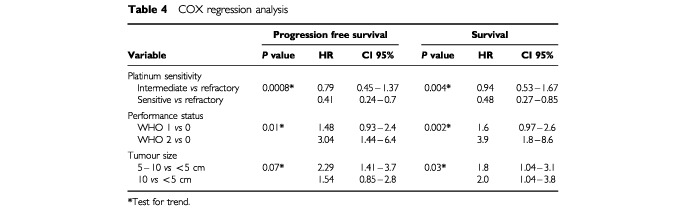
COX regression analysis

). In the Cox regression analysis, PFI since the last platinum-based chemotherapy, performance status and tumour size were statistically significant prognostic factors for both the OS and PFS, except for tumour size, which did not reach statistical significance for PFS (Table 4).

## DISCUSSION

In patients with ovarian cancer who relapse following platinum-based combination chemotherapy, the use of reinduction therapy, again consisting of a platinum-based regimen is considered effective only if the interval between the completion of platinum therapy and recurrence is at least 12 months. In patients who relapse within 12 months following the completion of therapy, reinduction platinum-based therapy is generally considered not appropriate, and alternative options including the use of new active agents have been widely adopted as standard of care. The key reference to substantiate that effective reinduction therapy with platinum is restricted to patients with PFIs of more than 12 months is the study reported by Markman *et al* (1991). In that study the response rate to salvage platinum chemotherapy was 50% in patients with an interval of ⩾12 months following completion of prior therapy, whereas patients with briefer intervals responded in less than 30% of cases. In the subgroup of patients who had relapsed during or within 4 months after completion of therapy, further treatment with platinum was found ineffective. Consequently, based upon the treatment-free interval patients have been classified as potentially platinum-sensitive (>12 months interval), intermediate-sensitive (4–12 months), and -refractory (relapsing during or within 4 months after platinum therapy). In recent consensus classifications these categories still exist and are widely used to determine appropriate therapy for individual patients (Berek *et al*, 1998). Of note, these results, as well as those reported in more recent studies were obtained with conventional platinum-based therapy with either cisplatin or carboplatin administered every 3 or 4 weeks.

Thus far, there have been limited data to support the use of weekly cisplatin salvage therapy in patients with ovarian cancer relapsing after conventional platinum-based therapy (Bolis *et al*, 1994).

We previously reported several studies investigating the feasibility and the efficacy of weekly cisplatin plus daily etoposide in patients with solid tumours (Planting *et al*, 1995). In view of the favourable results obtained in these studies, we initiated the current phase II study in patients with ovarian cancer relapsing after platinum-based chemotherapy. We began our study in patients who had a treatment-free interval of more than 12 months, who received cisplatin at a dose of 50 mg m^−2^ weekly plus daily etoposide. With increasing experience we amended the protocol to also include patients with treatment intervals less than 12 months, who were scheduled to receive cisplatin at a dose of 70 mg m^−2^ weekly plus daily etoposide. Despite prior treatment with platinum compounds, with 22% of the patients having received two and 3% of the patients having received even three prior platinum containing regimens, we found that non-haematologic toxicity, particularly neurotoxicity and renal toxicity was notably mild.

Haematologic toxicity resulted in several treatment postponements and dose reductions, but the median dose intensity of cisplatin achieved in patients scheduled to receive 50 mg m^−2^ days 1, 8, 15 and 29, 36 and 43 (equaling 37.5 mg m^−2^ week^−1^) was 32 mg m^−2^ week^−1^, and in patients scheduled to receive 70 mg m^−2^ (calculated dose per week 52.5 mg m^−2^) it was 45 mg m^−2^. Haematologic complications were few, with no more than one episode of neutropenic fever.

We found this regimen of weekly cisplatin plus daily etoposide highly effective. Of the 38 cisplatin-sensitive patients 92% responded, with 63% complete responses, a response duration and PFS exceeding 1 year and OS exceeding 2 years. We also observed considerable effectiveness in patients considered only intermediately platinum-sensitive. In this group of 32 patients we obtained 91% responses, including 31% CR, a response duration of 9 months, PFS of 8 months, and an OS of 16 months. Even in the group of 28 patients who were considered platinum-refractory, with a median time since last platinum treatment of only 0.7 months (range 0–3.7 months), we still obtained 46% responses, including 29% CR, a response duration of 7 months, PFS of 5 months, and OS of 13 months. These results compare strikingly favourable to those previously reported by Markman *et al* (1991), and others (Seltzer *et al*, 1985; Gershenson *et al*, 1989; Kavanagh and Nicaise, 1989; van der Burg *et al*, 1991; Kjorstad *et al*, 1992), using reinduction therapy with 3- or 4-weekly intervals. Of note, cisplatin at a dose of 70 mg m^−2^ weekly six times in a period of 8 weeks, compared with the standard dose of 75 mg m^−2^ every 3 weeks results in a 2.1-fold higher schedule-intensity.

The criterion of treatment-free interval since last platinum therapy is also indicative for the response rate to other agents. The newly adopted agents such as paclitaxel and docetaxel, topotecan, caelyx and gemcitabine, although effective in patients with treatment-free intervals of more than 12 months, are infrequently producing responses in patients who are considered cisplatin-refractory. Response rates with these new agents in this subgroup of patients are consistently 15% or less, and even in patients considered intermediate-sensitive to platinum retreatment, these new compounds in monotherapy give response rates of no more than 20–30%, with a median PFS of 3–5 months at best (Eisenhauer *et al*, 1994; Kohn *et al*, 1994; Lund *et al*, 1994; Kavanagh *et al*, 1996; Muggia *et al*, 1997; ten Bokkel Huinink *et al*, 1997; Tropé *et al*, 1998; Gordon *et al*, 2000).

The greater majority of our patients in this study had received their initial chemotherapy in the era that the platinum-paclitaxel combination had not yet been introduced. As a consequence, the majority of our patients had received one or two regimens consisting of cisplatin plus cyclophosphamide every 3 weeks, or carboplatin plus cyclophosphamide every 3 or 4 weeks. As the purpose of our study was to investigate the potential of schedule-intensive cisplatin in patients who had previously been treated with conventionally scheduled platinum therapy we do not believe that the main clinical finding reported in this study with weekly cisplatin plus etoposide would be different if paclitaxel had been substituted for cyclophosphamide in the first line regimen.

In view of these results, we believe that unless toxicity from previous therapy prohibits the use of cisplatin, all patients with ovarian cancer irrespective of the platinum-free interval should be considered for a rechallenge with therapy containing weekly cisplatin. In this study we report on weekly cisplatin plus daily etoposide. Weekly cisplatin can be combined with other agents that are active in ovarian cancer, and we are currently investigating the potential of the combination of weekly cisplatin plus weekly paclitaxel (van der Burg *et al*, 1998).

We conclude that schedule-intensive treatment with weekly cisplatin plus daily oral etoposide is highly effective in patients with ovarian cancer relapsing after conventional platinum-based combination chemotherapy, including patients who have progressed during or relapsed within 4 months after the completion of treatment. The optimal use of schedule intensive therapy incorporating new active agents, such as paclitaxel, is currently being investigated.
